# Commentary: The Impact of Digital Technology on Psychological Treatments and Their Dissemination

**DOI:** 10.3389/fpsyg.2018.01571

**Published:** 2018-08-28

**Authors:** Alexandre Heeren

**Affiliations:** ^1^Psychological Science Research Institute, Université Catholique de Louvain, Louvain-la-Neuve, Belgium; ^2^Institute of Neuroscience, Université Catholique de Louvain, Louvain-la-Neuve, Belgium

**Keywords:** evidence-based psychological treatment, clinical psychological treatment, mental healthcare, digital treatment, computerized therapy, mental health, digital technology, treatment dissemination

Fifty years ago, psychological treatments of mental health problems were beginning to undergo a radical shift. The research methods of experimental psychology and experimental clinical medicine began to be applied to the development and assessment of psychological treatments. Now, psychological treatments are beginning to undergo a new shift, driven by the widespread availability of digital technology. Taking stock of the current tech revolution occurring in clinical psychology, Fairburn and Patel (2017) provided a much-needed narrative review of the digital interventions to date (i.e., mobile apps, internet-based interventions, computerized cognitive training), those in the pipeline, and their likely impact on clinical practice and the global dissemination of psychological treatments.

I congratulate Fairburn and Patel (2017) for engaging in such a comprehensive review about the digital transformation of psychological treatments. I am particularly enthusiastic about the way the authors pave the way for best anticipating the digital transformation of psychological treatments. I also share the view that digital training via massive online open courses and other online platforms are key leverage points to progressively foster the global dissemination of evidence-based psychological treatments. However, as appealing and intriguing as the enthusiasm of the authors may sound, I argue that the authors overestimated the current evidence regarding the beneficial impact of digital treatments *per se*.

First, despite encouraging evidence regarding the effectiveness of internet-based intervention for a broad range of mental disorders (e.g., Andrews et al., 2010; Riper et al., 2014), both the quality and the effectiveness of most digital interventions remain unclear. For instance, the vast majority of mental-health mobile apps and therapist-free computerized training are neither theory-driven nor evidence-based (Donker et al., 2013; Anthes, 2016). Some of these apps may even be harmful (e.g., Gajecki et al., 2014; Anthes, 2016). Although supporting evidence is building (e.g., Dagöö et al., 2004; Birney et al., 2016), much of the research has been limited to pilot studies, often conducted by the apps' own developers rather than by independent researchers, and randomized clinical trials tend to be statistically underpowered and unreplicated (Anthes, 2016; Torous et al., 2017). And, with respect to the very few theory-driven and evidenced-based therapist-free computerized treatments, such as the cognitive bias modification and cognitive training procedures, their effectiveness remains extremely limited, suggesting that they are not yet ready for global dissemination (Cristea et al., 2015; Heeren et al., 2015, 2016; Firth et al., 2017). Given the pace at which digital mental-health companies are blooming and mobile apps are being released on app stores, theoretically grounded and methodologically robust research studies evaluating their efficacy and safety are promptly needed before endorsing their dissemination. Features like double-blinding, adequate randomization, appropriate sample sizes, and reproducibility by independent researchers that have been long been required in the testing of new treatments (e.g., Moher et al., 1998, 2009; Yordanov et al., 2015) are only now starting to find their way into the research on the digital treatment. As such, in contrast to Fairburn and Patel (2017) who strongly advocate for the global dissemination of those new treatments, I believe that both clinical scientists and digital mental-health companies should first improve the methodological quality of the research related to those new treatments.

Second, beyond research quality, another critical point to consider relates to the treatment adherence. Indeed, dropout rates are significantly higher for digital treatments^1^ than usual face-to-face ones (e.g., Christensen et al., 2009; Kelders et al., 2012; van Ballegooijen et al., 2014). As such, it raises questions about adherence to digital treatments. Although it is unknown whether these patients dropped-out as a result of the intervention or because they get worse and cannot be followed-up (Holmes et al., 2018; Karyotaki et al., 2018), nonadherence constitutes a significant barrier that should imperatively be considered before promoting the global dissemination of digital treatments, especially given the existence of a strong link between treatment adherence and outcomes (e.g., Kane, 2007; Donkin et al., 2011). Of critical importance, nonadherence increases risk for chronification and development of subsequent health problems (e.g., Martin et al., 2005). In this way, nonadherence also carries a huge economic burden, with yearly expenditures resulting from nonadherence estimated to be in the hundreds of billions of US dollars (e.g., Martin et al., 2005; Cutler et al., 2018). Although nonadherence can take many forms (e.g., misunderstanding of the instructions, oversight, or complete ignorance of the program; Martin et al., 2005), one common explanation for the nonadherence to digital treatments focuses on the absence of therapeutic alliance (e.g., Sucala et al., 2012; Strand et al., 2017). Although positive therapeutic alliance can be established when interactions with a professional via e-mail, chat technology, or video are included in the digital treatments (e.g.,Mohr et al., 2011; Berger, 2017), much of the digital interventions available on the market are completely free from such interactions. In contrast to Fairburn and Patel (2017) who advocate to focus on global dissemination, I am thus encouraging to first focus on treatment adherence rather than global dissemination. Particularly, efforts should be made to develop sound theoretical and empirical frameworks on the development of treatment adherence for digital treatments (Mohr et al., 2011). Yet uncertainty remains about the optimal way to maximize treatment adherence in therapist-free digital treatments.

Altogether, although I agree with Fairburn and Patel (2017) that an audit of the impact of digital technology on psychological treatments and their dissemination is timely, I call for a reconsideration of their enthusiasm regarding the current evidence associated with the efficacy of digital treatments. For all the aforementioned points, I think that it is urgently critical to first foster the improvement of both the research quality and treatment adherence regarding those new digital treatments before advocating for their global dissemination.

## Author contributions

The author confirms being the sole contributor of this work and approved it for publication.

### Conflict of interest statement

The author declares that the research was conducted in the absence of any commercial or financial relationships that could be construed as a potential conflict of interest.

## Footnotes

^1^Note that most of these studies focused on internet-based interventions and that there is almost no evidence regarding other types of digital treatments.
